# Anabolic Steroid Use for Weight and Strength Gain in Critically Ill Patients: A Case Series and Review of the Literature

**DOI:** 10.1155/2018/4545623

**Published:** 2018-05-07

**Authors:** Matthew Anstey, Shilpa Desai, Luke Torre, Bradley Wibrow, Jason Seet, Emma Osnain

**Affiliations:** ^1^Sir Charles Gairdner Hospital, Nedlands, WA, Australia; ^2^School of Medicine, University of Western Australia, Crawley, WA, Australia

## Abstract

**Background:**

An important long-term complication of critical illness is significant weakness and its resulting functional impairment. Recent advances have aimed to prevent critical illness weakness via early mobilisation of patients, minimising sedation, and optimising nutrition. One other potential treatment may be to provide anabolic support in the recovery phase, especially as patients have decreased levels of anabolic hormones.

**Case Presentation:**

We describe a case series of 4 patients who had either (1) profound critical illness myopathy and (2) profound weight loss. All patients were already receiving appropriate nutritional support and physiotherapy. All patients had functional improvements in their muscle strength.

**Conclusions:**

For patients in the recovery phase of critical illness, we provide examples of when anabolic steroid supplementation may assist the treating clinicians in rehabilitating their patients who are still in the Intensive Care Unit. We discuss patient selection and the current supporting literature for anabolic supplementation in critically ill patients.

## 1. Background

An important long-term complication of critical illness is significant weakness and its resulting functional impairment [1]. The mechanisms leading to profound weakness are diverse but include catabolism due to caloric deficits, increased stress hormone production, prolonged immobility, and inflammation causing myoneuropathy [2]. These result in structural changes that include axonal nerve degeneration, muscle myosin loss, and muscle necrosis [3]. Recent advances have aimed to prevent critical illness weakness via early mobilisation of patients, minimising sedation, and optimising nutrition [2, 4–6]. One other potential treatment may be to provide anabolic support in the recovery phase [4]. Low testosterone levels are frequent in critically ill patients [5, 7, 8]. This may reflect hormonal dysregulation in acute illness, as is seen with other hormones (insulin, cortisol). However, hypotestosteronaemia after acute illness may contribute to impaired recovery and rehabilitation. Testosterone supplementation in older healthy men has been shown to have benefits on physical functioning, including improved fat-free mass and muscle strength [9, 10].

We describe a case series of patients for whom we prescribed a course of anabolic steroids for one of the following indications: (1) profound weakness or (2) significant weight loss. All patients were already receiving sufficient nutritional support (at least 3 days of caloric intake at estimated goal) and were engaging in physiotherapy (graded rehabilitation according to optimal mobility scale) [11].

Anabolic steroids have two main properties: androgenic and anabolic effects. The different mechanisms include modulation of androgen receptor expression and interference of glucocorticoid receptor expression, which results in an anticatabolic effect. The most common androgen is testosterone, which exerts its effects by directly binding with the androgen receptors. Testosterone also undergoes metabolism via the aromatase or 5-*α* reductase enzymes to convert to oestradiol and 5-*α*-dihydrotestosterone, respectively. Many synthetic or designer anabolic steroids have had modifications to the testosterone structure to maximise the anabolic properties, while attempting to eliminate the androgenic effects. Such changes enhance protein anabolic effects and thus present themselves as potential therapeutic options for the restoration of fat-free muscle mass, and strength in chronic illnesses or conditions including critical illness related myopathy. In our first patient, we used testosterone due to an inability to obtain nandrolone. Testosterone possesses a relatively balanced ratio of myotrophic to androgenic activity (0.7–1.3), which means more virilizing effects for women, potential aggression, and negative impact on cholesterol levels. Danazol is an isoxazole of testosterone with weak androgenic activity and no oestrogenic activity. Alternative anabolic agents such as nandrolone or oxandrolone exhibit significantly greater selectivity for myotropic properties, with minimal androgenic effects (myotrophic : androgenic activity ratio of 12 and 13, resp.) [12]. Consequently, the potential for adverse outcomes including aromatization and virilizing effects in women is minimised.

Patients were considered for participation if they had profound weight loss or muscle loss as a result of their illness or Intensive Care Unit (ICU) admission and were in the recovery phase and failing to make progress. Consent to receive the medication was obtained from the patient, and an individual patient approval was obtained for our hospital pharmacy. All patients consented to publication of these results.  Table 1 outlines the indications and results of anabolic steroid supplementation for these patients.

## 2. Case Presentations

### 2.1. Patient 1

A 23-year-old lady was admitted to the ICU with respiratory distress requiring emergent intubation on 25 March 2014. She was being managed on the ward for bilateral infected leg ulcers, on a background of systemic lupus erythematous (SLE), hypertension, rheumatic fever, chronic renal failure, microscopic haemolytic anaemia, and extensive oesophageal ulceration. Her admission weight was 60.5 kg.

The cause of her respiratory failure was invasive aspergillosis and CMV pneumonitis. This was further complicated by microangiopathic haemolytic anaemia and thrombocytopenia thrombotic purpura requiring plasmapheresis, liver dysfunction, and renal failure requiring continuous renal replacement therapy. A percutaneous tracheostomy was performed on day 10 of her ICU admission. By day 22, she had lost > 25% weight from admission despite provision of enteral nutritional support aimed at 120% of predicted caloric requirements. She was clinically severely deconditioned (serum albumin 22) with evidence of fat and muscle wasting, undergoing a protracted wean from the ventilator (on day 30 she was still receiving pressure support ventilation 12/5 and transitioned to T-piece ventilation trials on day 31), and globally weak with 1-2/5 power throughout.

She was commenced on testosterone (Sustanon®) 250 mg IM on day 32. Subjectively her appetite improved as did her ventilator weaning and she was decannulated 3 days after the first dose. After further consultation with the immunology team, the Sustanon was replaced by 200 mg danazol daily from day 43 due to improved safety in SLE. Upon discharge to the ward (day 46), her upper limb strength had improved to 3-4/5 bilaterally and 2/5 power in the lower limbs. She was able to move from supine to standing with minimal assistance and continued with tilt table for strengthening and stretching. After 3 weeks of rehabilitation on the ward, she could walk approximately 20 m unassisted with a frame. She was subsequently transferred to a rehabilitation unit on day 62 and discharged home on day 73, after 30 doses of danazol.

### 2.2. Patient 2

A 62-year-old male presented to the Emergency Department on 14 June 2015 with fevers and rigors, 5 days after a complex transoesophageal AF ablation (admission weight 139 kg). Following a brief cardiac arrest on the coronary care unit, a CT chest revealed an oesophageal perforation and atrio-oesophageal fistula. He underwent urgent repair of the left atrio-oesophageal fistula. Postoperatively he developed mediastinitis and vasopressor dependent shock. On day 8, he developed severe abdominal pain and had a right hemicolectomy and ileostomy for caecal perforation. Following these complications, he was severely deconditioned (critical illness myopathy was evident from day 9) and received a tracheostomy on day 16. Despite optimal nutritional supplementation, he was losing weight (at one point he lost 6 kg weight in one week, and from admission weight to time of administration he had lost 16.6% of admission weight) with evidence of fat and muscle wasting. Due to the profound weakness in all limbs, an electromyogram (EMG) was performed on day 39, which was consistent with severe critical illness myopathy and mild axonal degeneration neuropathy.

He was prescribed nandrolone 50 mg IMI on day 82. At this point, despite daily physiotherapy throughout his stay, he required full hoist transfers. At the time of his second injection (day 90) his strength was dramatically improving with 3-4/5 power throughout. Serial testosterone measurements decreased from 10 before first dose to 5.7 before the second dose and 7.3 before the third dose. Although he still required full hoist for transfers, he could perform up to 6 reps of sitting to standing with ArjoHoist®. He was also able to sit unassisted for 30 minutes. On day 95 he was transferred to a rehabilitation unit and received his third dose of nandrolone the following day. He eventually flew back to Queensland (his home) and received ongoing rehabilitation and strengthening at a local hospital.

### 2.3. Patient 3

A 49-year-old male was admitted on 7 April 2015 with worsening abdominal pain on a background of known diverticulitis and colovesical fistula. After an initial anterior resection and cystectomy, he returned to theatre a further 5 times due to anastomotic breakdown and faecal peritonitis. He also developed multiorgan failure as a result of septic shock.

During his ICU stay, he lost approximately 16 kg (17% of admission weight) over 2 months, despite adequate parenteral and enteral feeding. In addition, he had overwhelming critical illness weakness with severe limb weakness. On day 29 after admission, he required pressure support ventilation via his tracheostomy and had only a flicker of movement in his limbs distally. He was given 50 mg IMI of nandrolone. One week later prior to receiving his second dose, he no longer required ventilatory assistance, and his power was measured at 2/5 on the left side of his body and 2+ on the right. He received subsequent doses on days 36 and 48.

At day 46, he was sitting on the edge of the bed with assistance and on day 48 he could walk a few assisted steps with a frame. His tracheostomy was removed on day 51. He was discharged from ICU on the 2 June (54 days after admission) with muscle power measured at 4/5 bilaterally (3/5 ankle/toes). On discharge from hospital on day 85 he was able to independently move, including up and down a flight of stairs.

### 2.4. Patient 4

A 70-year-old male, with a background of colonic cancer resection (1997) and recurrent small bowel obstructions (SBO), was admitted on 10 July 2015 with another SBO. During this admission, his weight dropped to 42 kg (BMI 13.5 kg/m2) with evidence of severe fat and muscle wasting. The weight loss was attributed to severe malnutrition related to chronic poor oral intake. He was commenced on total parenteral nutrition (TPN) after failed enteral feeding. Dexamethasone and mirtazapine were trialled as appetite stimulants, but he developed further obstruction which was managed conservatively.

He was severely deconditioned and had not been able to move out of bed for the first 20 days of admission due to profound weakness. He was not receiving mechanical ventilation. He was severely fatigued on transfer to the commode or shower and had 3/5 power throughout his limbs. He was able to ambulate less than 10 m with a frame. Nandrolone 50 mg IMI was prescribed to assist predominantly with weight gain, but also with deconditioning. He received his first dose on day 60 and a subsequent dose on day 69. He gained 4.5 kg following his first dose and was able to ambulate independently for gradually increasing short distances with a frame. He was discharged on day 89 with home TPN and rehabilitation in the home.

## 3. Discussion and Conclusions

In each of the cases presented, despite attempts to optimise their nutrition and physical rehabilitation, there was limited improvement. As a result, the treating team chose to trial anabolic steroids as an adjunct to standard care. The loss of lean body mass in critical illness is associated with misalignment between catabolic and anabolic hormones. Correcting this imbalance with hormonal therapy such as anabolic steroids could increase weight and improve nitrogen balance, respiratory muscle strength, and potential survival. It is important to note that the types of patients described, although not frequent in the Intensive Care Unit, do have extremely long stays in the ICU and use a large number of resources. Reducing that burden has significant implications for the ICU, its staff, and the patient.

For all of the patients involved, we were careful to ensure that the patient was in the recovery phase of illness. We defined this as no ongoing organ support (such as needing vasopressors or inotropes), with no increasing inflammatory markers. Low testosterone levels, while frequently seen, may actually serve as an energy conservation mechanism for the body under stress. In addition, we are aware that most patients in the recovery phase after critical illness will regain their muscle strength, especially with the addition of early mobility programs, but this can be an extremely slow process [11, 14, 15]. For this reason, we would also consider using anabolic steroids to assist with patients suffering a protracted ventilator wean.

As a caution, the literature suggests that there may be several contraindications to hormonal supplementation in the critically ill, apart from the listed contraindications (prostate cancer, liver disease, pregnancy, prepubescent). A previous randomised controlled trial using growth hormone found an increased mortality [16]. It may be that, during the state of critical illness, hormonal supplementation may be proinflammatory or misdirects protein synthesis to protein building rather than immune function. Furthermore, an observational study suggests an association between testosterone supplementation and increased cardiac events [17]. Just as athletes have used performance enhancing supplements in conjunction with strength training and nutritional supplementation, it is essential that our patients have sufficient nutritional stores to build muscle and that they are able to interact with a rehabilitation program. Thus, we selected our patients for anabolic steroid therapy if they were stable, which was defined as having no evidence of ongoing sepsis (no fevers, increasing inflammatory markers, or broad spectrum antibiotics) or heart disease (no recent myocardial infarction and ejection fraction > 35%); myopathic; adequately awake to participate in rehabilitation/physiotherapy; and receiving sufficient nutrition to make muscle.

The choice of dose was based on a literature review. Nandrolone has been used in a variety of doses and settings. Outpatient HIV patients with HIV wasting syndrome were given 150 mg fortnightly in one study and 200/400/600 mg weekly in another study for 12 weeks. In both studies the intervention was associated with weight gain [18, 19]. A randomised comparison of nandrolone, testosterone, or placebo for adult HIV patients with HIV wasting syndrome showed superior weight gain and improvement in BMI and quality of life from nandrolone [20]. Patients with COPD were given 50 mg fortnightly for 4 doses with improvement of muscle function and exercise capacity. Patients with chronic renal failure on haemodialysis were given nandrolone (100 mg females, 200 mg males) and undertook resistance training weekly for 6 months with improvement in functional activities (walking and stair-climbing), but no difference in grip strength between groups [21]. The study closest to our case series was a pilot study giving 50 mg of nandrolone weekly for 3 weeks to critically ill patients which resulted in positive nitrogen balance in 8/10 patients [22]. Interestingly, in patient 2 we were able to measure sequential testosterone levels, which actually showed a decrease in levels, which has been previously shown, as nandrolone may reduce endogenous testosterone production [20].

Our results need to be interpreted with caution. There are multiple factors at play in the recovery of a critically ill patient, such as nutritional supplementation, ongoing low grade illness, dose of physical rehabilitation, and the patient's baseline health. While most of our outcome indicators suggest an improvement, it may be that these achievements would have happened regardless of the anabolic supplementation. For instance, patient 1 was undergoing a protracted ventilatory wean but was making some progress and while the anabolic supplementation may have accelerated this, it is also possible it made no difference. Changes in weight in these patients may reflect fluid shifts (especially in those on dialysis), and lean body mass or estimation of muscle thickness may have been better outcome variables [23]. Handgrip strength is independently associated with poor hospital outcome and serves as a simple test to identify ICU acquired weakness, but with asymmetric weakness it may not be the best measure [13, 24]. Grip strength did not improve in all our patients. Furthermore, timing of administration of anabolic supplementation is important, as administering it too early before neuronal injury has improved may prevent optimal results. The duration of therapy may also be of importance and most of the outpatient studies gave the therapy for a longer duration, which we elected not to do, as the patients were no longer in our unit. However, we did not detect any significant adverse effects of the anabolic supplementation. Finally, there was one other patient who we considered for a course of anabolic steroids, but after the first dose and recurrence of sepsis we elected not to give ongoing doses.

Critical illness weakness is an important patient outcome that is associated with medical, economical, and psychosocial adverse outcomes for the patient, the patient's family, and the community [2]. There are limited medical interventions that have shown promise in this important condition. This case series suggests a possible role for stimulation of muscle growth using anabolic steroids. Amongst long stay ICU survivors, it is estimated that one-quarter to one-half or more will suffer from significant weakness and impaired mobility and function. While the absolute numbers of patients this affects is low, the consequences of a prolonged ICU stay, deconditioning, and muscle loss have significant implications on their ability to return to a functional quality of life [25, 26]. We have commenced a pilot randomised controlled trial to explore these results further (the GAINS trial ACTRN12616000835448), including ultrasound measurements of quadriceps thickness as a surrogate for muscle strength as an outcome and functional activity levels using the Chelsea critical care physical assessment tool [23, 27].

## Figures and Tables

**Table 1:** Overview of indications and results of anabolic steroid supplementation.

	Indication for anabolic steroids	Date of first dose administered	Testosterone level before administration	Weight change after administration	Grip strength change	Strength change
Patient 1	Profound critical illness weakness	Day 32 of ICU stay	0.6 nmol/L (<2.0 nmol/L)	+*7.3 kg* 45 kg (22/4) → 52.3 (15/5)	(L) +4.4 kg (R) +6 kg	*From* 1/5 power globally *to* ambulation with minimal assist on discharge

Patient 2	Profound critical illness weakness	Day 82 of ICU stay	5.7 nmol/L (1035 nmol/L)	−*3.8 kg* 115.8 kg (3/9) → 112 (17/9)	(L) −0.6 kg (R) +5.7 kg	*From* profound generalised weakness (0-1/5) *to* multiple repetitions of assisted sitting to stand exercises and tracheostomy decannulation

Patient 3	Profound critical illness weakness	Day 30 of ICU stay	Not available	+*1.5 kg* 72.5 kg (23/5) → 74 kg (26/5)	Grip strengths not assessed	*From* profound weakness (flicker contraction 1/5) *to* increased strength in all muscle groups (3-4/5)

Patient 4	Long-term malnutrition and inability to gain weight or strength	Day 70 of admission and ICU review for ongoing TPN/nutritional advice	6.5 nmol/L (10–35 nmol/L)	+*4.4 kg*	(L) −3.6 kg (R) −3.4 kg	Power globally 3/5. *From* fatigue on transfers *to* ambulating independently for very short distances with frame

Handgrip threshold of 11 kg force in males and 7 kg force in females for the diagnosis of ICU acquired weakness (sensitivity 80.6%, specificity 83.2%, NPV 92.3%, and PPV 63.0%) [13].

## Abbreviations

ICU: Intensive Care Unit
SBO: Small bowel obstruction
SLE: Systemic lupus erythematous.
